# Bone Marrow-Derived Mesenchymal Stem Cells Ameliorate Sepsis-Induced Acute Kidney Injury by Promoting Mitophagy of Renal Tubular Epithelial Cells *via* the SIRT1/Parkin Axis

**DOI:** 10.3389/fendo.2021.639165

**Published:** 2021-06-25

**Authors:** Jun Guo, Rong Wang, Donghai Liu

**Affiliations:** Department of Critical Medicine, Union Jiangbei Hospital, Huazhong University of Science and Technology, Wuhan, China

**Keywords:** sepsis-induced acute kidney injury, bone marrow mesenchymal stem cell, mitophagy, Parkin, SIRT1, apoptosis, pyroptosis

## Abstract

Sepsis is a common risk factor for acute kidney injury (AKI). Bone marrow-derived mesenchymal stem cells (BMSCs) bear multi-directional differentiation potential. This study explored the role of BMSCs in sepsis-induced AKI (SI-AKI). A rat model of SI-AKI was established through cecal ligation and perforation. The SI-AKI rats were injected with CM-DiL-labeled BMSCs, followed by evaluation of pathological injury of kidney tissues and kidney injury-related indicators and inflammatory factors. HK-2 cells were treated with lipopolysaccharide (LPS) to establish SI-SKI model *in vitro.* Levels of mitochondrial proteins, autophagy-related proteins, NLRP3 inflammasome-related protein, and expressions of Parkin and SIRT1 in renal tubular epithelial cells (RTECs) of kidney tissues and HK-2 cells were detected. The results showed that BMSCs could reach rat kidney tissues and alleviate pathological injury of SI-SKI rats. BMSCs inhibited inflammation and promoted mitophagy of RTECs and HK-2 cells in rats with SI-AKI. BMSCs upregulated expressions of Parkin and SIRT1 in HK-2 cells. Parkin silencing or SIRT1 inhibitor reversed the promoting effect of BMSCs on mitophagy. BMSCs inhibited apoptosis and pyroptosis of RTECs in kidney tissues by upregulating SIRT1/Parkin. In conclusion, BMSCs promoted mitophagy and inhibited apoptosis and pyroptosis of RTECs in kidney tissues by upregulating SIRT1/Parkin, thereby ameliorating SI-AKI.

## Introduction

Sepsis can be a fatal organ dysfunction resulted from the host’s response to infection, representing the most frequent cause of acute kidney injury (AKI) in critical patients (1, 2). Sepsis-induced acute kidney injury (SI-AKI) is not only a major problem in medical, surgical and intensive care unit (ICU), but also an independent risk factor for high mortality and increased hospitalization and cost (3). The clinical features of SI-AKI are oliguria and declined kidney solute clearance (4) which usually results in electrolyte and acid-base disorders, fluid overload, and toxic accumulation of metabolites and drugs (5). Notably, the elderly populations are particularly vulnerable to sepsis, and the incidence rate of SI-AKI tends to continue to rise along with the global aging trend (6). At present, there is no specific targeted treatment or intervention measure, with kidney replacement therapy remaining the basic available option for severe cases (3). Hence, further elucidating the molecular mechanism of SI-AKI is the urgent issue to be solved to open up novel therapy targets for SI-AKI.

Energy metabolism dysfunction is a key factor that contributes to the pathogenesis of AKI, and the effect of mitochondria in AKI has been extensively investigated since it works as the center of energy metabolism (7). Selective degradation of damaged or depolarized mitochondria is known as mitophagy, which is indispensable for maintaining mitochondrial quality control and intracellular homeostasis (8). Recently, the critical role of mitophagy in the progression of AKI and subsequent kidney repairing has been unveiled (9). For instance, Rong et al. have revealed that enhancement of mitophagy effectively protects the kidney from the tubular epithelial cells injury induced by iohexol (10). Similarly, mitophagy may also represent a promising therapeutic target for SI-AKI.

Bone marrow-derived mesenchymal stem cells (BMSCs) possess self-renewal and multi-differentiation, which play critical roles in tissue maintenance, repairing and regeneration (11). BMSCs have been demonstrated to exert effective therapeutic effects on AKI (12). BMSCs can localize to kidney chambers and promote kidney regeneration by differentiation or paracrine action, which bear broad application prospects in the clinical treatment of AKI benefited from the advantages of low toxicity and autotransplantation (13). Xu et al. have exhibited that BMSC injection can reduce mortality, improve lung injury and decline levels of pro-inflammatory factors in mice with cecal ligation and puncture (CLP) induced sepsis (14). However, whether BMSCs ameliorate SI-AKI by regulating mitophagy remains unclear. In view of this, we investigated the potential protective mechanism of BMSCs in SI-AKI by regulating mitophagy.

## Materials and Methods

### Ethics Statement

The study got the approval of the Ethical Committee of Union Jiangbei Hospital, Huazhong University of Science and Technology. All experimental procedures were implemented on the Ethical Guidelines for the study of experimental pain in conscious animals.

### Isolation and Identification of BMSCs

Female Sprague–Dawley rats aged 12 weeks (15) and weighed 260–300 g were obtained from the Second Affiliated Hospital of Guangzhou Medical University [SYXK (Guangdong) 2018-0192]. Rats were injected with excessive phenobarbital, and then the bilateral femur and tibia were removed. The bone cavity was rinsed with Dulbecco’s modified Eagle’s medium/Ham’s nutrient mixture F-12 (DMEM/F12) (Gibco, Grand Island, NY, USA). BMSCs (5 × 10^6^ cells/ml) were seeded in DMEM/F12 containing 10% fetal bovine serum (FBS) (Sigma-Aldrich, Merck KGaA, Darmstadt, Germany) and 10 ng/ml epidermal growth factor (Peprotech, Offenbach, Germany). BMSCs were cultured, passaged and purified by adherence method.

BMSCs of passage 3 in logarithmic growth period were collected. The surface antigens CD45 (ab10558, Sigma-Aldrich), CD34 (ab81289, Sigma-Aldrich), CD29 (ab36219, Sigma-Aldrich) and CD90 (ab225, Sigma-Aldrich) of BMSCs were detected using a flow cytometer. The osteogenic and adipogenic ability of BMSCs was detected by adipogenic and osteogenic induction solution. Adipogenic induction solution contained DMEM-HG, 10% FBS, 1 μmol dexamethasone, 0.5 mmol 3-isobutyl-1-methylxanthine, 10 μg/ml insulin and 0.2 mmol indomethacin. Osteogenic induction solution contained DMEM-HG, 10% FBS, 1 μmol dexamethasone, 10 mmol β-glycerophosphate and 50 mg/L vitamin C. After 2 weeks of adipogenic induction, cells were rinsed with phosphate-buffered saline (PBS) three times, fixed with 10% paraformaldehyde for 10 min, stained with oil red O for 5–10 min, washed with 60% isopropanol to remove excess dye solution, stained with hematoxylin, differentiated with 1% hydrochloric acid, and rinsed with water for 10 min, and then adipogenesis was observed. After 3 weeks, the osteoblast induction solution was sucked away, and then cells were rinsed with PBS for three times, and stained with alizarin red for 10–15 min, and then osteogenesis was observed.

### Animal Grouping

CLP of rats was performed by reference to a previous literature (16). The rats were anesthetized with 60 mg/kg pentobarbital and placed on Homeothermic Blanket Control Unit (507220F, Harvard Apparatus) to keep the body temperature at 36.537°C. A small longitudinal midline incision was made to expose the cecum, and then the cecum was ligated at 1 cm from the end of the cecum. In the direction of ligation to the cecum, a puncture was performed between the ligation part and the tip of the cecum to perforate the cecum. After removing the needle, a small drop of feces was squeezed from the two perforated holes to ensure unobstructed. In sham operated rats, the cecum was not ligated or perforated. After 12 h of CLP treatment, rats were euthanized with 4% pentobarbital to collect kidney tissue (17, 18), blood, and urine for analysis. After successful modeling, survival analysis was carried out. The rats were observed for 7 days and the survival rate of each group was calculated. The animal experiment was strictly complied with the Guiding Opinions on Treating Experimental Animals Well [(2006) No. 398] issued by the Ministry of Science and Technology of the People’s Republic of China, and followed the Guidelines for the Protection and Use of Laboratory Animals issued by National Institutes of health (No.85-23, 1996 revision). Extensive efforts were made to minimize the number of animals and reduce the suffering of included animals.

Totally 96 rats were assigned into six groups: sham group (CLP sham operation was performed), CLP group (3 h after CLP operation, rats were injected with 0.2 ml normal saline *via* tail vein), CLP + BMSCs group [3 h after CLP operation, rats were injected with 0.2 ml normal saline containing 1 × 10^6^ BMSCs *via* tail vein; BMSCs were labeled on ice with 4 mg/L chloromethyl-benzamide dialkylcarbocyanine (CM-Dil) (Invitrogen, Carlsbad, CA, USA) for 15 min before injection], CLP + BMSCs + siParkin group (48 h before CLP operation, rats were injected with 5 × 10^7^ TU/ml Parkin silencing lentivirus *via* tail vein; 3 h after CLP operation, rats were injected with 0.2 ml normal saline containing 1 × 10^6^ BMSCs *via* tail vein), CLP + BMSCs + siNC group (48 h before CLP operation, rats were injected with 5 × 10^7^ TU/ml Parkin silencing lentivirus blank control *via* tail vein), and CLP + BMSCs + EX527 group [3 h after CLP operation, rats were injected with 0.2 ml normal saline containing 1 × 10^6^ BMSCs and 10 mg/kg SIRT1 inhibitor EX527 (Selleck) *via* tail vein] (**Figure S1**). Lentivirus siParkin and siNC were purchased from Shanghai GENECHEM Co., Ltd. (Shanghai, China). Six rats in each group were used for experimental verification, and the other six rats were used for survival rate detection.

### Histological Analysis

Kidney tissues were fixed with 4% paraformaldehyde, embedded in paraffin and sliced at 4 μm. The paraffined sections were dewaxed, rehydrated and stained with hematoxylin & eosin (HE) (Beyotime Biotechnology Co., Ltd, Shanghai, China), followed by observation under a microscope (CKX41SF, Olympus, Tokyo, Japan). Morphological changes were evaluated according to the acute tubular necrosis (ATN) scoring system adopted by Dragun (2001; magnification ×200, each kidney section quantified by ATN scoring system ≥20 visual fields). The evaluation of histopathological changes included the absence of renal tubular brush border, dilatation of renal tubules, mold formation, and cell lysis. The tissue injury was quantified using blind method and scored according to the percentage of injured tubules in the sample: 0, no injury; 1, <25% injury; 2, 25–50% injury; 3, 50–75% injury; 4, >75% injury (19).

### Immunohistochemistry

The paraffined sections were dewaxed, dehydrated with gradient ethanol, and washed with PBS (three times/3 min). Then, the sections were cultured with 3 ml/L methanol-H_2_O_2_ for 15 min to block endogenous peroxidase, followed by PBS washing (three times/3 min). Next, the sections were detached with 1 g/L trypsin at 37°C for 30 min to expose intracellular antigens, followed by PBS washing (three times/3 min). After that, the sections were blocked with skim milk powder and cultured with the primary antibodies: anti-NLRP3 (ab214185, 1/100, Abcam Inc., Cambridge, MA, USA), anti-ACS (ab177958, 1/200, Abcam), anti-caspase-1 (ab74279, 1/250, Abcam), and anti-IgG (ab172730, 1/500, Abcam) at 4 °C overnight. Following PBS washing (three times/3 min), the sections were cultured with the secondary antibody immunoglobulin G (IgG) (ab6721, 1/1000, Abcam) at 37°C for 1 h. Thereafter, the sections were developed with 2,4-diaminobutyric acid (DAB) and counterstained with hematoxylin for 3–5 min. Afterwards, the sections were dehydrated, dried and sealed.

### Localization of BMSCs in Kidney Tissues

The kidney tissues embedded in optimal cutting temperature compound were made into frozen sections and washed with PBS three times. Then, the sections were stained with 4’, 6-Diamidino-2-phenylindole dihydrochloride (DAPI) for 5 min to label nuclei. The distribution of BMSCs in kidney tissues was observed under a confocal laser microscopy.

### Observation of Renal Tubular Epithelial Cells (RTECs) in Kidney Tissues

The observation of RTECs was performed by reference to a previous literature (20). Briefly, the kidney cortex of rats in each group (Sham, CLP, CLP + BMSCs, CLP + BMSCs + siNC, CLP + BMSCs + siParkin-LV, and CLP + BMSCs + EX527 groups) was sliced into pieces and cultured with 1 mg/ml type-I collagenase at 37°C for 30 min. The red blood cells were lysed and removed. RTECs were obtained by Percoll gradient density centrifugation for Western blotting and related experiments.

### TUNEL Assay

After deparaffination and dehydration, the paraffined sections were stained using the TUNEL apoptosis assay kit (Beyotime), and developed with DAB. Then, five fields of vision were randomly selected to count number of TUNEL-positive cells.

### Enzyme-Linked Immunosorbent Assay (ELISA)

Blood urea nitrogen (BUN) and serum creatinine in the blood were detected using an automatic biochemical analyzer (AU680, Beckman Coulter, CA, USA). Levels of TNF-α in the serum and IL-1β and IL-6 in kidney tissues were detected using the ELISA kit (Jiancheng Bioengineering Institute, Nanjing, Jiangsu, China).

### Cell Culture and Grouping

HK-2 cells obtained from Sigma-Aldrich were incubated in DMEM/F12 containing 10% FBS at 37°C and 5% CO_2_ in a humidified atmosphere. HK-2 cells were treated with 10 mmol/L LPS (Sigma-Aldrich) to induce mitophagy.

HK-2 cells were assigned into six groups: blank group (HK-2 cells were incubated in normal conditions), LPS group (HK-2 cells were treated with LPS), LPS + BMSCs group (4 h after LPS treatment (21), HK-2 cells were co-cultured with 1 × 10^4^ BMSCs in 96-well Transwell plates), LPS + BMSCs + siNC group (4 h after LPS treatment, HK-2 cells were transfected with 50 nM siNC and cultured with 1 × 10^4^ BMSCs in 96-well Transwell plates), LPS + BMSCs + siParkin group (4 h after LPS treatment, HK-2 cells were transfected with 50 nM siParkin and cultured with 1 × 10^4^ BMSCs in 96-well Transwell plates) and EX527 group (4 h after LPS treatment, HK-2 cells were supplemented with 10 uM EX527 (22, 23) and co-cultured with 1 × 10^4^ BMSCs in 96-well Transwell plates). Cell transfection was conducted in line with the instructions of Lipofectamine™ 2000 reagent (Invitrogen). The above siRNA plasmids were purchased from GENECHEM (Shanghai, China).

### Immunofluorescence Staining

A total of 1 × 10^6^ cells were seeded into the culture dish, washed with PBS, and permeabilized with PBS containing 0.1% Triton X-100 and 0.1% sodium citrate at 4°C. Next, the samples were blocked with 10% goat serum albumin for 1 h and cultured at 4°C overnight with the primary antibodies Parkin (1/1,000, Cell Signaling Technology, Beverly, MA, USA) and Sirt1 (1/1,000, Cell Signaling Technology). Following PBS washing three times, the samples were incubated with Alexa Fluor 488 donkey anti-rabbit antibody (1/1,000, Invitrogen) for 1 h. Cells were observed under an inverted fluorescence microscope at 40× magnification (BX51, Olympus).

### Measurement of Mitophagy

pCMV-GFP-LC3 plasmids (American Type Culture Collection, Manassas, Virginia, USA) were transfected into HK-2 cells using the GeneJammer reagent (Agilent Stratagene, Palo Alto, CA, USA). HK-2 cells stably expressing GFP-LC3 were cultured with 150 umol/L palmitic acid for 24 h and then treated with 50 nM red-fluorescing MitoTracker Red (M22426, Invitrogen) for 30 min. Thereafter, cells were fixed with 4% paraformaldehyde, permeabilized with 0.1% Triton X-100 and observed under a fluorescence microscope.

### Western Blotting

Total protein was extracted in radio-immunoprecipitation assay buffer (strong) (Beyotime) and the concentration of proteins was tested using the bicinchoninic acid assay kit (Beyotime). Next, the proteins were separated by electrophoresis and transferred onto polyvinylidene fluoride membranes. The membranes were blocked with 5% skim milk and washed by tris buffered saline tween (TBST). Afterwards, the membranes were cultured with the primary antibodies at 4°C overnight: Parkin (ab77924, 5 µg/ml, 52k Da, Abcam), SIRT1 (ab110304, 1–0.125 µg/ml, 110 kDa, Abcam), TOM20 (ab186735, 1/1,000, 16 kDa, Abcam), TIM23 (ab230253, 1/1,000, 22 kDa, Abcam), LC3 (ab192890, 1/2,000, 15 kDa, Abcam), P62 (ab109012, 1/10,000, 15 kDa, Abcam), p-P62 (ab211324, 1/1,000, 47 kDa, Abcam), Bax (ab32503, 1/10,000, 21 kDa, Abcam), Bcl-2 (ab59348, 1/500, 26 kDa, Abcam), cleaved caspase-3 (ab49822, 1/500, 17 kDa, Abcam), NLRP3 (ab263899, 1/1,000, 118 kDa, Abcam), ASC (ab175449, 1–3 µg/ml, 21 kDa, Abcam), caspase-1 (ab179515, 1/1,000, 42 kDa, Abcam), KIM-1 (Cat#PA5-20244, 1/500, 51 kDa), and GAPDH (ab8245, 1/500, 36 kDa, Abcam). After being washed by TBST (three times/10 min), the membranes were cultured with secondary antibody horseradish peroxidase-conjugated goat anti-rabbit IgG H&L (1/2,000, ab205718, Abcam) for 1 h and then washed by TBST (three times/10 min) before chemiluminescence developing and visualization. The image of protein blotting was analyzed by Image J2x v2.1.4.7 software (Rawak Software, Inc. Dresden, Germany).

### Statistical Analysis

Data analysis was introduced by the SPSS 21.0 (IBM Corp., Armonk, NY, USA). Kolmogorov–Smirnov method checked the data were in normal distribution. Data are expressed as mean ± standard deviation. One-way analysis of variance (ANOVA) was employed for the comparisons among multiple groups, and Tukey’s multiple comparisons test was utilized for the *post hoc* test after ANOVA. The *p* value was obtained from a two-tailed test, and *p <*0.05 meant a statistically significance.

## Results

### Isolation and Identification of BMSCs

**Figure 1:**
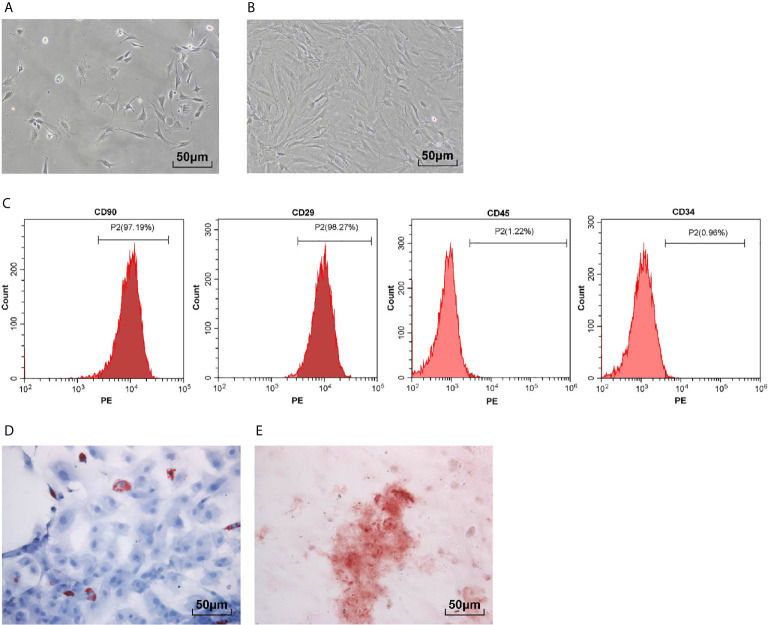
Isolation and identification of BMSCs. **(A)** Representative images of primary BMSCs; **(B)** Representative images of BMSCs at passage 3; **(C)** Surface antigens (CD45, CD34, CD29 and CD90) of BMSCs at passage 3 were detected using flow cytometry; **(D)** BMSCs were stained with oil red O after adipogenic induction; **(E)** BMSCs were stained with alizarin red after osteogenic differentiation induction. Each experiment was repeated for three times independently.

The results of light microscope showed that there were a certain amount of impurity cells in primary cultured BMSCs (**Figure 1A**). The impurity cells in BMSCs almost disappeared after three generations of culture (**Figure 1B**). The surface antigens (CD45, CD34, CD29 and CD90) of BMSCs at passage 3 were detected using flow cytometry. The CD29- and CD90-positive rates were 98.1 and 97.5%, and the CD45- and CD34-positive rates were 1.31 and 1.01%, respectively (**Figure 1C**). Oil red O staining and alizarin red staining demonstrated that the isolated BMSCs had the adipogenic and osteogenic differentiation ability (**Figures 1D, E**). It was suggested that BMSCs were isolated successfully.

### BMSCs Alleviated SI-AKI in Rats

**Figure 2:**
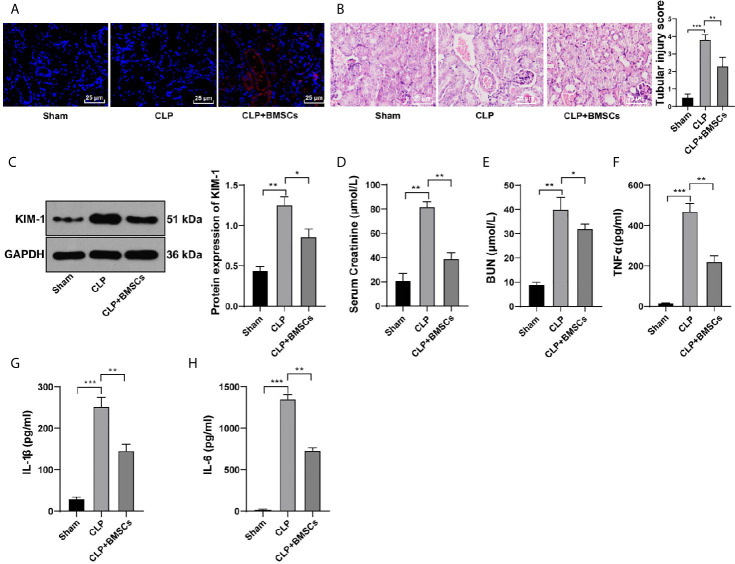
BMSCs alleviated SI-AKI in rats. **(A)** Distribution of CM-Dil-labeled BMSCs in kidney tissues was observed under a confocal laser microscopy; **(B)** Renal tubular injury was evaluated using HE staining; **(C)** Expression of KIM in kidney tissues was detected using Western blotting and quantitatively analyzed; **(D, E)** Levels of BUN and serum creatinine in the blood were detected; **(F–H)** Levels of TNF-α in serum, and IL-1β and IL-6 in kidney tissues were detected by ELISA. N = 6. Each experiment was repeated for three times independently. Data are presented as mean ± standard deviation and analyzed using one-way ANOVA, followed by Tukey’s multiple comparison test for the *post hoc* test, ^*^ *p < *0.05, ^**^ *p < *0.01, ^***^ *p < *0.001.

CM-Dil can bind to the surface of living cells and show stable red fluorescence. We used CM-Dil-labeled BMSCs to determine whether BMSCs could reach the kidney tissues and affect kidney injury. We found that obvious red fluorescence occurred in the kidney tissues of rats injected with CM-Dil-labeled BMSCs *via* tail vein (**Figure 2A**), indicating that BMSCs could reach the kidney tissues of rats.

A previous literature has revealed the protective role of BMSCs in kidney injury (24). Hence, we investigated whether BMSCs could improve SI-AKI in rats. Our results showed that BMSCs ameliorated the loss of the brush border, cast formation and vacuolization, and decreased the index of kidney tubular injury of rats with SI-AKI (**Figure 2B**; *p <*0.01). KIM-1 expression in kidney tissues, the biomarker of kidney tubular injury, was detected. The results showed that BMSCs notably reduced the expression of KIM-1 in kidney tissues (**Figure 2C**; *p <*0.05). Additionally, BMSCs deceased the levels of BUN and serum creatinine in the blood (**Figures 2D, E**; *p <*0.05), and reduced the contents of inflammatory factors in serum and kidney tissues (**Figures 2F–H**; *p <*0.05). All these results suggested that BMSCs could alleviate SI-AKI in rats.

### BMSCs Promoted Mitophagy of HK2 Cells and RTECs in Kidney Tissues in Rats With SI-AKI

**Figure 3:**
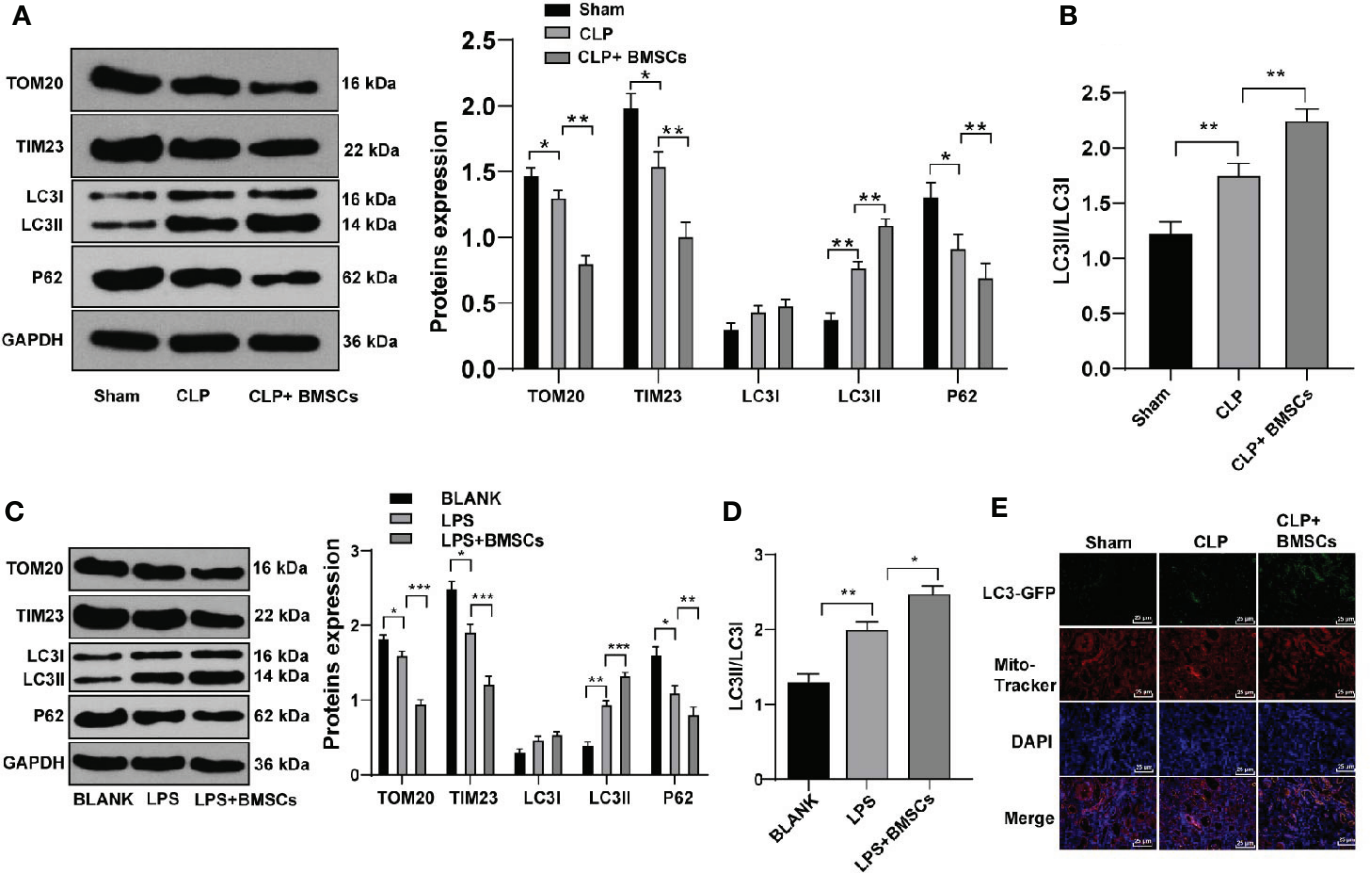
BMSCs promoted mitophagy in RTECs of kidney tissues and HK-2 cells. **(A)** Levels of mitochondrial proteins and autophagy-related proteins in kidney tissues were detected using Western blotting; **(C)** Levels of mitochondrial proteins and autophagy-related proteins in HK-2 cells were detected using Western blotting; **(B, D)** Ratio of LC3II/LC3I in kidney tissues and HK-2 cells were detected using Western blotting; **(E)** Mitophagy was detected using immunofluorescence staining. Each experiment was repeated for three times independently. Data are presented as mean ± standard deviation and analyzed using one-way ANOVA, followed by Tukey’s multiple comparison test for the *post hoc* test, ^*^ *p < *0.05, ^**^ *p < *0.01, ^***^ *p < *0.001.

It is reported that AKI activates mitophagy of RTECs, and mitophagy protects RTECs (25). We hypothesized that the protective effects of BMSCs on SI-AKI was induced by promoting mitophagy in RTECs. Therefore, we detected the levels of mitophagy-related proteins in RTECs in kidney tissues. Compared with that in the model group, the rats in the BMSCs group showed decreased levels of TOM20 and TIM23, increased ratio of LC3II to LC3I, and decreased level of p62 (**Figures 3A, B**; all *p <*0.05).

Meanwhile, HK-2 cells were treated with LPS to simulate SI-AKI *in vitro*. Similarly, HK-2 cells in the BMSCs group showed decreased levels of TOM20 and TIM23, increased ratio of LC3II to LC3I, and decreased level of p62 (**Figures 3C, D**; all *p <*0.05). Additionally, LC3-GFP-labeled autophagosomes co-localized with Mito-Tracker-labeled mitochondria in HK-2 cells was increased after BMSCs treatment (**Figure 3E**). It was suggested that BMSCs promoted mitophagy in RTECs of rats with SI-AKI.

### BMSCs Promoted Mitophagy of HK2 Cells and RTECs in Kidney Tissues of Rats With SI-AKI by Increasing Parkin

**Figure 4:**
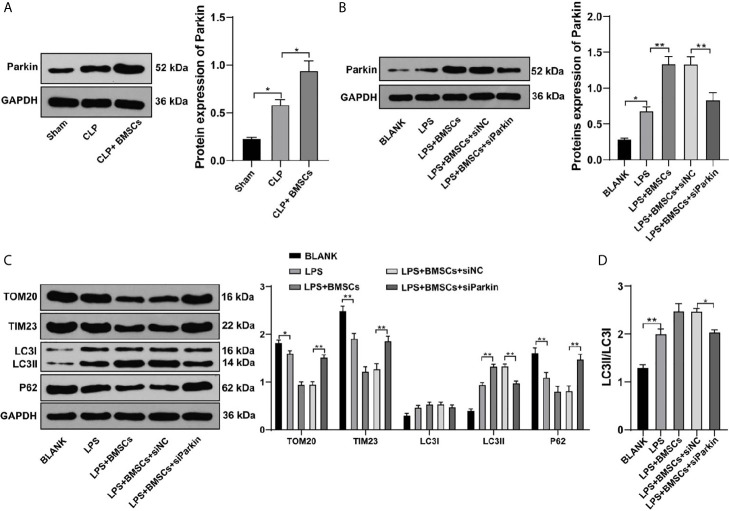
BMSCs promoted mitophagy in RTECs of kidney tissues and HK-2 cells by upregulating Parkin. **(A, B)** Expression of Parkin in kidney tissues and HK-2 cells was detected using Western blotting; **(C)** Effects of LPS, BMSCs and siParkin on the levels of mitochondrial proteins (TOM20 and TIM23) and autophagy-related proteins (LC3II, LC3I, and P62) in HK-2 cells were detected using Western blotting; **(D)** Ratio of LC3II to LC3I detected using Western blotting. Each experiment was repeated for three times independently. Data are presented as mean ± standard deviation and analyzed using one-way ANOVA, followed by Tukey’s multiple comparison test for the *post hoc* test, ^*^ *p < *0.05, ^**^ *p < *0.01.

Parkin-mediated mitophagy has critical influences on RTECs injury (26). We found that Parkin expression was increased slightly in RTECs in kidney tissues of SI-AKI rats and in LPS-treated HK-2 cells, while BMSCs could notably upregulate Parkin expression (**Figures 4A, B**; *p <*0.05). Transfection of siParkin reversed the decreases of TOM20, TIM23 and P62, as well as the increase of LC3II/LC3I induced by BMSCs (**Figures 4C, D**; all *p <*0.05). These results showed that BMSCs promoted mitophagy of rats with SI-AKI by upregulating Parkin expression.

### BMSCs Promoted Mitophagy of RTECs in Kidney Tissues and HK2 Cells *via* SITR1/Parkin

**Figure 5:**
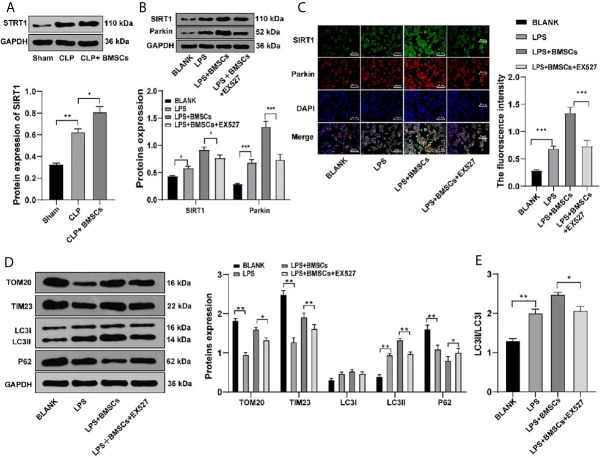
BMSCs promoted mitophagy in RTECs of kidney tissues and HK-2 cells *via* SITR1/Parkin. **(A, B)** SIRT1 expression of RTECs in kidney tissues was detected using Western blotting; **(C)** Expressions of SIRT1 and Parkin in HK-2 cells were detected using immunofluorescence staining; **(D)** Levels of mitochondrial proteins (TOM20 and TIM23) and autophagy-related proteins (LC3II, LC3I, and P62) in HK-2 cells were detected using Western blotting; **(E)** Ratio of LC3II to LC3I was detected using Western blotting. Each experiment was repeated for three times independently. Data are presented as mean ± standard deviation and analyzed using one-way ANOVA, followed by Tukey’s multiple comparison test for the *post hoc* test, ^*^ *p < *0.05, ^**^ *p < *0.01, ^***^ *p < *0.001.

SIRT1 plays a vital role in Parkin-mediated mitophagy (27), and MSCs can regulate the expression of SIRT1 in cardiomyocytes (28). Therefore, we speculated that BMSCs might regulate Parkin expression *via* SIRT1, thus affecting mitophagy. Our results showed that BMSCs could upregulate SIRT1 expression in RTECs in rat kidney tissues and in HK-2 cells (**Figures 5A, B**; *p <*0.05).

We detected Parkin expression in HK-2 cells co-treated with EX527 (a SIRT1 inhibitor) and BMSCs to determine whether SIRT1 was involved in the regulation of Parkin by BMSCs. We found that EX527 could inhibit the increase of Parkin expression induced by BMSCs (**Figure 5B**; *p <*0.001). The results above were verified using immunofluorescence assay (**Figure 5C**). Moreover, EX527 could reduce the promotion of mitophagy induced by BMSCs (**Figures 5D, E**; *p <*0.05). It was indicated that BMSCs regulated Parkin expression *via* SIRT1 and then promoted mitophagy.

### BMSCs Inhibited Apoptosis of RTECs in Kidney Tissues of Rat With SI-AKI *via* the SITR1/Parkin Axis

**Figure 6:**
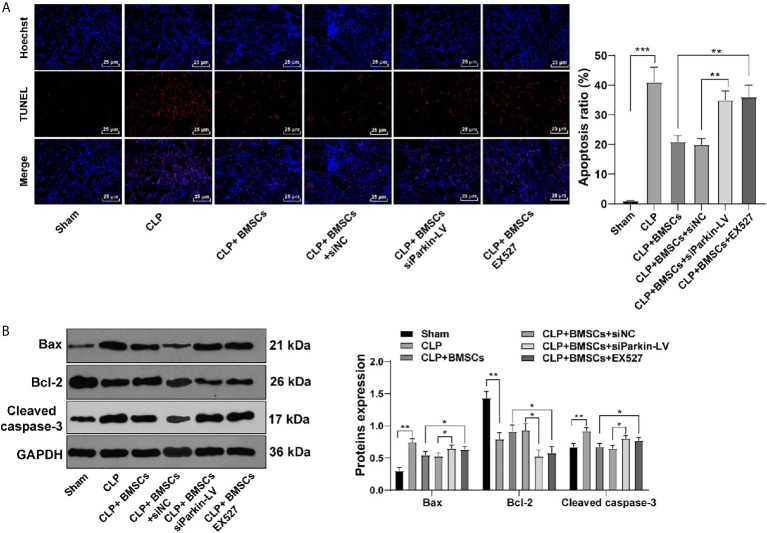
BMSCs inhibited apoptosis of RTECs of rats with SI-AKI *via* the SITR1/Parkin axis. **(A)** Apoptosis of RTECs in kidney tissues of rats was measured using TUNEL staining; **(B)** Levels of apoptosis-related proteins of RTECs in kidney tissues of rats were detected using Western blotting. Each experiment was repeated for three times independently. Data are presented as mean ± standard deviation and analyzed using one-way ANOVA, followed by Tukey’s multiple comparison test for the *post hoc* test, ^*^ *p < *0.05, ^**^ *p < *0.01, ^***^ *p < *0.001.

Mitochondrial-mediated apoptosis exerts significant effects on AKI (29). We detected the levels of apoptosis-related proteins (Bax, cleaved caspase-3 and Bcl-2) in RTECs in kidney tissues of SI-AKI rats to investigate the role of BMSCs promoting mitophagy in apoptosis. BMSCs inhibited RTECs apoptosis of rats with SI-AKI, with decreased Bax and cleaved caspase-3, and increased Bcl-2. Parkin knockdown or EX527 reversed the inhibitory effect of BMSCs on apoptosis of RTECs (**Figures 6A, B**; all *p <*0.01). These results indicated that BMSCs inhibited apoptosis of RTECs of rats with SI-AKI *via* the SITR1/Parkin axis.

### BMSCs Inhibited Pyroptosis of RTECs in Kidney Tissues of Rats With SI-AKI *via* the SITR1/Parkin Axis

**Figure 7:**
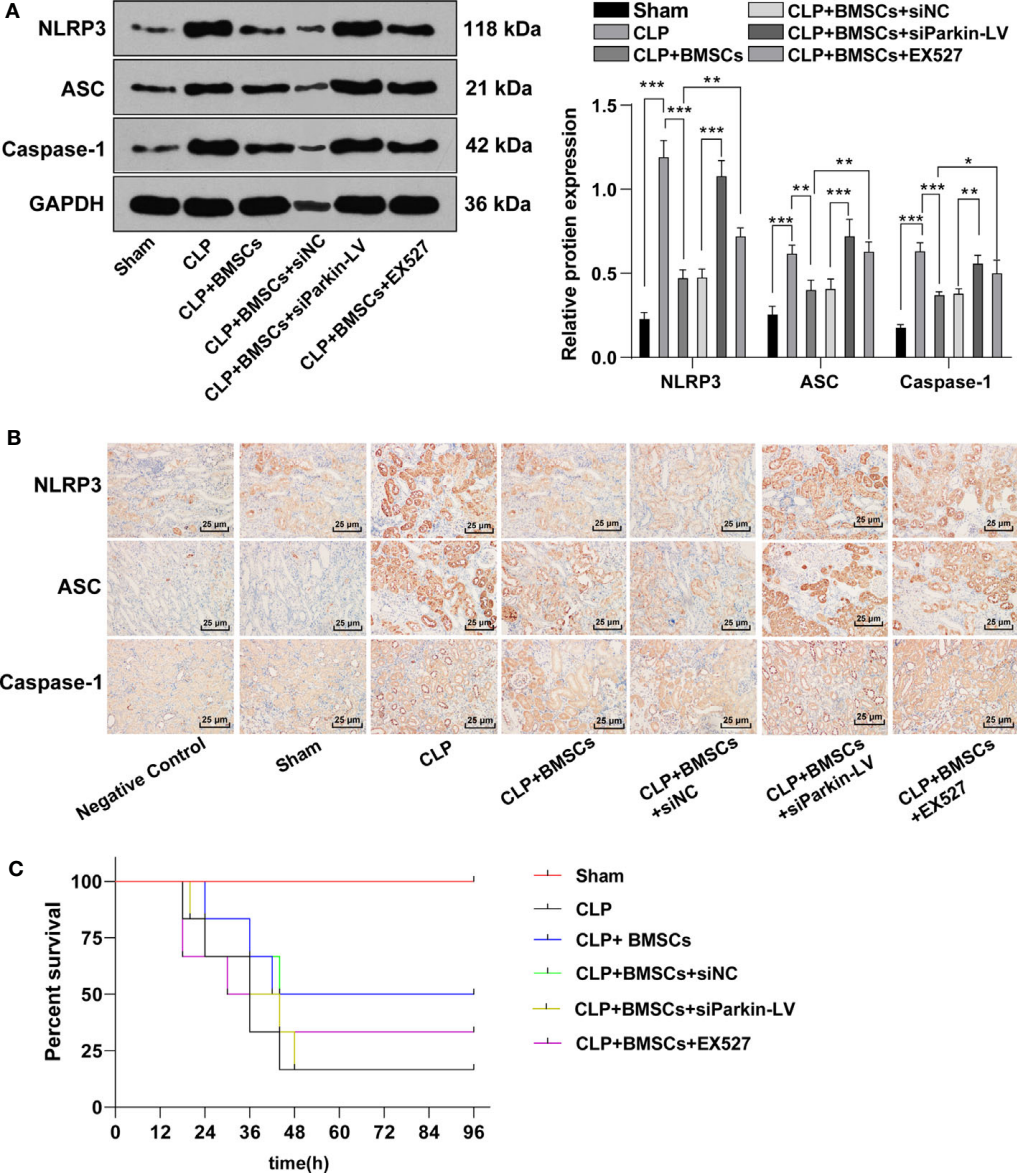
BMSCs inhibited pyroptosis of RTECs of rats with SI-AKI *via* the SITR1/Parkin axis. **(A)** Levels of inflammasome activation related-proteins (NLRP3, ASC and caspase-1) in RTECs of rats were detected using Western blotting; **(B)** Levels of inflammasome activation related-proteins (NLRP3, ASC and caspase-1) in RTECs of rats were detected using immunohistochemistry. **(C)** Survival rate of rats in each group. Each experiment was repeated for three times independently. Data are presented as mean ± standard deviation and analyzed using one-way ANOVA, followed by Tukey’s multiple comparison test for the *post hoc* test, ^*^ *p < *0.05, ^**^ *p < *0.01, ^***^ *p < *0.001.

NLRP3-mediated pyroptosis may aggravate kidney injury (30), and mitophagy inhibits the activation of NLRP3 (31). The increased levels of NLRP3, ASC and caspase-1 led to the activation of inflammasome, and then promoted pyroptosis. We detected the levels of NLRP3, ASC and caspase-1 in RTECs in kidney tissues of SI-AKI rats to evaluate the role of BMSCs promoting mitophagy in pyroptosis. The levels of NLRP3, ASC and caspase-1 were elevated in rats with SI-AKI, indicating that SI-AKI promoted pyroptosis of RTECs in kidney tissues; BMSCs inhibited pyroptosis of RTECs in kidney tissues of rats with SI-AKI. Parkin knockdown or EX527 treatment could reverse the inhibitory effect of BMSCs on pyroptosis of RTECs (**Figures 7A, B**; *p <*0.05). The survival rate of sham-operated rats was 100%. CLP decreased the survival rate of rats, while BMSCs treatment increased the survival rate. In addition, we also found that Parkin knockdown or SIRT1 inhibitor EX527 decreased the survival rate of rats (**Figure 7C**; *p <*0.05). These results suggested that BMSCs suppressed pyroptosis of RTECs in kidney tissues of rats with SI-AKI *via* the SITR1/Parkin axis.

## Discussion

SI-AKI is a prevalent clinical syndrome occurring in ICU patients with high mortality, and the survival rate after sepsis intricately relies on the recovery of kidney function (32). Intriguingly, the potential role of BMSCs in the management of SI-AKI has received increasing concerns (33). We demonstrated here that BMSCs had protective influences on SI-AKI by promoting mitophagy and inhibiting pyroptosis *via* the SIRT1/Parkin.

BMSC transplantation has been demonstrated to attenuate kidney injury and reduce mortality in AKI patients (34). For example, intrarenal injection of BMSCs can improve ischemia/reperfusion-induced AKI through anti-inflammatory and paracrine mechanism (35). BMSCs can notably increase the survival rate and recover the organ function of mice with CLP-induced sepsis (36). In this study, a rat model of SI-AKI was established using CLP method and then the rats were treated with BMSCs. The results of HE staining exhibited that BMSCs ameliorated the loss of the brush border, cast formation and vacuolization. KIM-1 is a transmembrane protein in injured proximal RTECs and serves as a prominent biomarker of kidney injury (37). We showed that BMSC treatment notably reduced the expression of KIM-1 in the kidney tissues of rats. The intensity of inflammation related to sepsis may reflect AKI severity and exert particular impacts on kidney injury (38). Liu et al. have shown that BMSC-based therapies improve gentamicin-induced AKI by facilitating the recovery of biochemical indicators in blood and inhibiting inflammation (12). Consistently, we exhibited that the inflammatory factor level in serum and kidney tissues of rats with SI-AKI were reduced after BMSC treatment. MSC treatment alleviates SI-AKI and improves the survival of mice with polymicrobial sepsis (39). All the results above indicated that BMSCs exerted protective effects on rats with SI-AKI.

Damaged mitochondria will eventually be degraded by mitophagy, and the disorder of mitophagy is concerned with the pathogenesis of AKI (40). Zhao et al. have revealed that enhancing mitophagy may protect rats against LPS-induced AKI (41). We hypothesized that the protective effects of BMSCs on SI-AKI was induced by promoting mitophagy of RTECs. Our experiments exhibited that BMSCs notably decreased the levels of mitochondrial proteins (TOM20 and TIM23), and increased autophagy-related proteins (LC3II/LC3I). Moreover, we treated HK-2 cells with LPS to establish SI-AKI model *in vitro*. The results *in vitro* were consistent with the results *in vivo*. BMSCs mediate a protection mechanism against sepsis by increasing mitophagy (36). Briefly, BMSCs could promote mitophagy.

Parkin, the E3 ubiquitin ligase, senses the functional status of mitochondria and marks the damaged mitochondria for disposal through autophagy pathway (42). Parkin-mediated mitophagy is implicated in the protective mechanism of polydatin on SI-AKI (43). We found that BMSCs could notably upregulate Parkin expression in rats with SI-AKI and in LPS-treated HK-2 cells. Knockdown of Parkin reversed the promoting effect of BMSCs on mitophagy. SIRT1 is a primary regulator directing the stress response to mitophagy (44). Elevated SIRT1 increases Parkin expression, thus leading to the activation of mitophagy (45). Our results revealed that BMSCs could upregulate SIRT1 expression in RTECs of rats and HK-2 cells. SIRT1 inhibitor could reverse the increase of Parkin and the promotion of mitophagy induced by BMSCs. Collectively, BMSCs regulated Parkin expression *via* SIRT1, and then promoted mitophagy.

Oxidative stress-mediated apoptosis of kidney tubular cells is recognized as a fundamental mechanism in kidney injury (29). Huo et al. have shown that inhibiting apoptosis and reducing inflammation may ameliorate diclofenac-induced AKI in mice (46). We exhibited that BMSCs inhibited RTEC apoptosis in rats with SI-AKI. Inhibition of apoptosis may be one of the mechanisms of BMSCs in repairing kidney injury (47). BMSCs can inhibit apoptosis of kidney cells and alleviate kidney injury caused by diabetic nephropathy (48). Moreover, NLRP3 inflammasomes are multi-protein heteromeric complexes that can response to cell injury or microbial infection, and assemble into ASC specks to activate caspase-1. Activation of inflammasomes induces secretion of pro-inflammatory cytokines and initiate pyroptosis (49, 50). NLRP3 inflammasome is associated with the pathogenesis of many kidney diseases, including AKI (51). Mitophagy negatively regulates the activation of NLRP3 inflammasome, thus preventing excessive inflammation caused by NLRP3 inflammasome activation (52). We found that BMSCs inhibited pyroptosis of RTECs of rats. BMSCs attenuate LPS-induced acute liver injury *via* suppressing NLRP3 inflammasome (53). Suppression of the NLRP3 pathway can protect mice against LPS-induced AKI (54). Additionally, we showed that knockdown of Parkin or SIRT1 inhibitor reverse the inhibitory effect of BMSCs on apoptosis and pyroptosis of RTECs. It is also reported that BMSCs can regulate SIRT1, thereby suppressing pyroptosis of myocardial cell and improving myocardial infarction (28). It was indicated that BMSCs suppressed apoptosis and pyroptosis of RTECs in rats with SI-AKI *via* SITR1/Parkin.

To sum up, BMSCs can promote mitophagy by upregulating SIRT1/Parkin, thereby protecting rats against SI-AKI. However, due to the current laboratory conditions, it is not possible to supplement the related experiments to characterize the phenotype of BMSCs in the kidney and the phenotype and secretory profile in the co-culture assays. In the future, we will carry out the related experiments if laboratory conditions and funding permit. Additionally, there are great differences in physiological structure between human and rats. We will also select animals closer to human relative for experiments to verify the effects of BMSCs on SI-AKI.

## Data Availability Statement

The raw data supporting the conclusions of this article will be made available by the authors, without undue reservation.

## Ethics Statement

The animal study was reviewed and approved by the Ethical Committee of Union Jiangbei Hospital, Huazhong University of Science and Technology.

## Author Contributions

All authors contributed to conceptualization, methodology, validation, formal analysis, investigation, resources, data curation, writing, review and editing, and visualization. All authors contributed to the article and approved the submitted version.

## Conflict of Interest

The authors declare that the research was conducted in the absence of any commercial or financial relationships that could be construed as a potential conflict of interest.
