# Supplementary figures and images for: Bone Marrow-Derived Mesenchymal Stem Cells Ameliorate Sepsis-Induced Acute Kidney Injury by Promoting Mitophagy of Renal Tubular Epithelial Cells via the SIRT1/Parkin Axis
Source: Front Endocrinol (Lausanne). 2021 Jun 25;12:639165. doi: 10.3389/fendo.2021.639165 (PMC8267935; doi:10.3389/fendo.2021.639165)

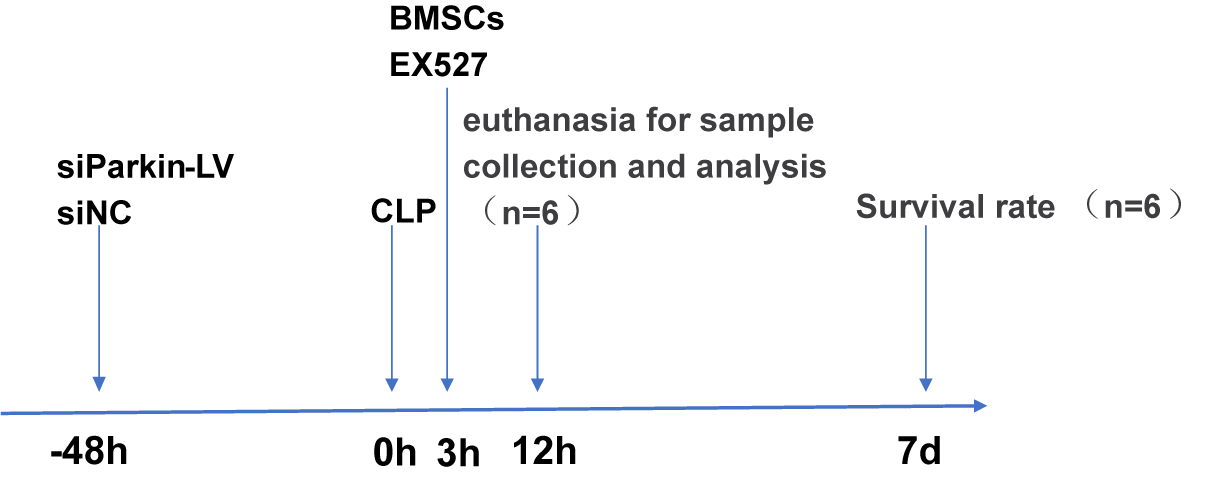

Supplement: Supplementary file 1 [file Image_1.tiff]
